# Revised recommendations from the CSO-HNS taskforce on performance of tracheotomy during the COVID-19 pandemic – what a difference a year makes

**DOI:** 10.1186/s40463-021-00531-z

**Published:** 2021-10-20

**Authors:** D. D. Sommer, D. Cote, T. McHugh, M. Corsten, M. A. Tewfik, S. Khalili, K. Fung, M. Gupta, N. Sne, P. T. Engels, E. Weitzel, T. F. E. Brown, J. Paul, K. M. Kost, J. A. Anderson, L. Sowerby, D. Mertz, I. J. Witterick

**Affiliations:** 1 Division of Otolaryngology - Head & Neck Surgery - Department of Surgery, McMaster University, Hamilton, ON Canada; 2 Division of Otolaryngology - Head and Neck Surgery, University of Alberta, Edmonton, AB Canada; 3 Division of Otolaryngology - Head & Neck Surgery, Dalhousie University, Halifax, NS Canada; 4 Department of Otolaryngology - Head and Neck Surgery, McGill University, Montreal, QC Canada; 5 Aurora Neuroscience Innovation Institute, Milwaukee, WI USA; 6 Department of Otolaryngology - Head and Neck Surgery, Western University, London, ON Canada; 7 Department of Surgery and Critical Care, McMaster University, Hamilton, ON Canada; 8 Department of Otolaryngology Head and Neck Surgery, Lackland Air Force Base, Wilford Hall Medical Center, San Antonio, TX USA; 9 Department of Anesthesia, McMaster University, Hamilton, ON Canada; 10 Department of Otolaryngology - Head & Neck Surgery, University of Toronto, Toronto, ON Canada; 11 Division of Infectious Disease, Department of Medicine, McMaster University, Hamilton, ON Canada

**Keywords:** Tracheotomy, Tracheostomy, COVID-19, SARS-CoV-2, Coronavirus, Intensive Care Unit/ICU, Critical Care, Mechanical Ventilation, Ventilator Weaning, Aerosol Generating Medical Procedure/AGMP, Percutaneous

## Abstract

**Background:**

During the early part of the COVID-19 pandemic, the Canadian Society of Otolaryngology - Head & Neck Surgery (CSO-HNS) task force published recommendations on performance of tracheotomy. Since then, our understanding of the virus has evolved with ongoing intensive research efforts. New literature has helped us better understand various aspects including patient outcomes and health care worker (HCW) risks associated with tracheotomy during the COVID-19 pandemic. Accordingly, the task force has re-evaluated and revised some of the previous recommendations.

**Main body:**

Based on recent evidence, a negative reverse transcription polymerase chain reaction (RT-PCR) COVID-19 swab status is no longer the main deciding factor in the timing of tracheotomy. Instead, tracheotomy may be considered as soon as COVID-19 swab positive patients are greater than 20 days beyond initial symptoms and 2 weeks of mechanical ventilation. Furthermore, both open and percutaneous surgical techniques may be considered with both techniques showing similar safety and outcome profiles. Additional recommendations with discussion of current evidence are presented.

**Conclusion:**

These revised recommendations apply new evidence in optimizing patient and health care system outcomes as well as minimizing risks of COVID-19 transmission during aerosol-generating tracheotomy procedures. As previously noted, additional evidence may lead to further evolution of these and other similar recommendations.

**Graphical abstract:**

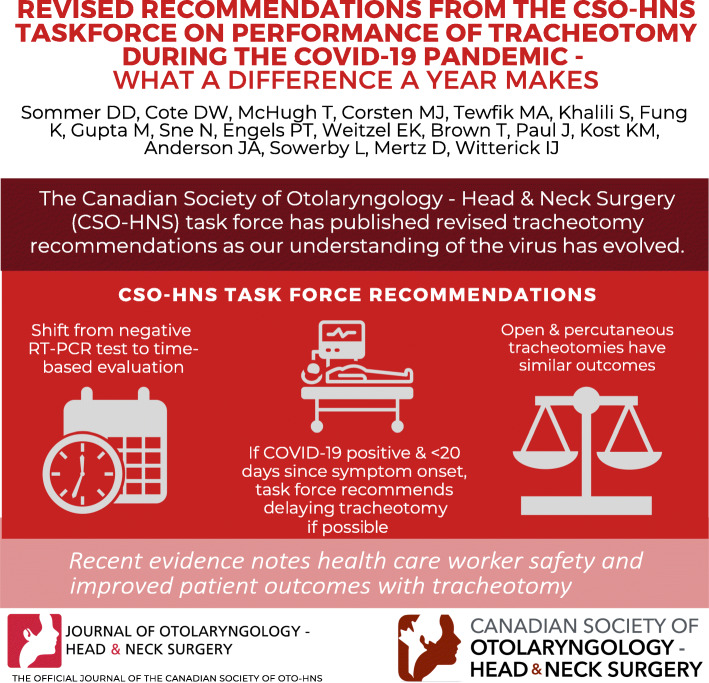

## Introduction

In April 2020, the CSO-HNS (Canadian Society of Otolaryngology-Head & Neck Surgery) taskforce published recommendations on performance of tracheotomy during the COVID-19 pandemic [1]. Tracheotomy is considered an AGMP (aerosol generating medical procedure) with significant risks of viral transmission [2–4]. Since then, there has been a growing body of evidence that has helped guide management decisions, however, some issues such as mutant viral strains continue to evolve while others remain unclear.

## Utility of performing tracheotomy in the critical care COVID-19 population

Recent systematic reviews have attempted to assess the utility of performing tracheotomy in the critically ill COVID-19 patient population [2, 4]. While these suggest some potential benefits in terms of decreased ICU (intensive care unit) stay and overall mortality, the level of heterogeneity and possible selection bias leave interpretation of these results open. There is likely a significant subgroup of patients with respiratory failure to wean and few other comorbidities that would potentially benefit from this intervention. Similarly, risks of tracheal/laryngeal injury with prolonged intubation of greater than 2–3 weeks need to be considered [5]. To date, there is insufficient evidence to suggest COVID 19 patients are substantially different in their risk for glottic injury than other ARDS (acute respiratory distress syndrome) prolonged intubated patients. Given the often delayed timing of tracheotomy in COVID 19 patients, it is possible that there is increased risk of laryngeal complications as a long-term sequelae [6].

## Changes to previous recommendations

### Defining COVID-19 positive status

The most significant changes in these recommendations pertain to reliance on a negative RT-PCR (reverse transcription polymerase chain reaction) test prior to proceeding with tracheotomy. In the earlier version of the recommendations, the consensus specified awaiting conversion to negative RT-PCR COVID-19 status. Since that time, several studies have described viral load dynamics as evidenced by a comparison of duration of viral shedding assessed by RT-PCR (viral fragments) vs detection of viable virus from time of symptom onset [7]. This evaluation of pooled data indicates that RT-PCR may remain positive for a mean of 17 days after onset of symptoms and has even been reported up to 83 days. Recent viral culture studies [8] have confirmed that viable virus has not been recovered in mild to moderate cases after 10 days, and in severe cases after 20 days with the risk of viral shedding dropping below 5% after 15.2 days [9]. Only rarely, in severely immunocompromised individuals, viable virus has been detected after 20 or more days [8, 10].

- We thus recommend a time-based evaluation of disease progression to define COVID-19 positivity and help guide decision making regarding performance of tracheotomy.
- Based on the recent CDC (Centers for Disease Control and Prevention) guidelines [8], a COVID-19 positive patient with severe to critical illness has been defined as cleared of the COVID-19 virus if it has been more than 20 days since (the earlier of) symptom onset or first positive diagnostic test. Prolonged RT-PCR can continue to be positive for up to 3 months after illness onset and does not necessarily denote transmissible disease and may represent inactive viral particles [9].

### COVID-19 RT-PCR positive (< 20 days)

- If a patient is COVID-19 positive and it has been less than 20 days since symptom onset or first positive RT-PCR, we recommend against performing a tracheotomy in this group of patients who are potentially still infectious [2, 3, 11, 12]. This should only generally be considered in this situation if the endotracheal tube is proving insufficient to provide an adequate airway, or an emergent procedure is required [1]. Enhanced PPE and environmental precautions are advised in these situations.
- In these patients, requests for tracheotomy should be considered in exceptional circumstances on a case-by-case basis with thorough discussion of the risks and benefits between the ICU staff and the attending surgeon.

### COVID-19 RT-PCR positive (> 20 days)

- If a patient is COVID-19 positive and it has been more than 20 days since symptom onset or first positive diagnostic test, a tracheotomy can be performed if otherwise clinically indicated.
- In general, patients undergoing consideration for tracheotomy should be mechanically ventilated/intubated for over 14 days.
- These patients may be considered “COVID-19 negative” for the purposes of tracheotomy, and the recommendations below should be followed. Post-operatively, these patients may be cared for as per local institution/regional protocols which may depend on COVID-19 RT-PCR testing with associated PPE and precautions.

### COVID-19 RT-PCR negative

- We are recommending (at minimum) N95 masks and full face/eye protection to be worn by the surgical team due to the possibility of false negative COVID-19 testing [13]. A negative result does not exclude the possibility of COVID-19 [14]. However, local epidemiology should be considered, and in time periods of low local epidemiology, the risk of a random patient to be unknowingly COVID-19 positive becomes negligible.
- Additionally, any other upper airway surgery that must proceed should have the requirement of COVID-19 testing/clearance of the patient before initiating surgery.

### COVID-19 unknown status

- For emergent tracheotomy with unknown COVID-19 status, our recommendations remain generally unchanged from previous. This includes the use of full aerosol PPE including N95 masks with full face/eye protection. Powered air purifying respirator (PAPR)/N99* equipment or equivalent is an option if available.
  - ○ *Although there is limited evidence to suggest possible superiority of PAPRs in limiting HCW exposures with other respiratory viruses (ie. influenza virus) as compared to N95 masks [15], there is currently a lack of field observational studies to suggest a difference in transmission risks to HCWs between the two types of PPE when performing AGMPs in patients with SARS-CoV-2 [16].
- Negative pressure rooms may be preferable if available.
- Intubation rather than tracheotomy is highly preferable if achievable.
- Caution is urged with the use of high flow oxygen/high flow nasal cannula, as well as unsealed non-invasive ventilation/bilevel positive airway pressure (BIPAP) as these are considered potential AGMPs and risk further transmission of disease [14].
- Intubation and/or tracheotomy should be performed by the most skilled person present to maximize initial attempt success [17], and minimize aerosolization risks.
- Awake tracheotomy and cricothyroidotomy are to be considered high risk for viral plume spread and should be avoided if possible. Only in extenuating circumstances should this be considered. A discussion between team members (e.g., anesthesia, otolaryngology, general/thoracic surgery, trauma team leader, emergency physician, critical care physician) should be undertaken to determine the risk/benefit profile for each situation.

### COVID-19 positive (> 90 days)

- If a patient is COVID-19 positive and it has been more than 3–6 months since initial positive result, there is the possibility of reinfection [8, 18, 19]. This may become an increasing issue with the emergence of new variants of SARS-CoV-2.
- In this situation, the patient should be retested for COVID-19 and if positive, the patient should be considered potentially re-infected, and if so, considered positive for < 20 days.
- If the patient is retested for COVID-19 and if negative, should be treated as COVID-19 negative.

### Health care team

- The recommendation to limit the number of team members during tracheotomy to reduce potential spread of disease remains. Furthermore, the surgical/anesthesia team providing care to the tracheotomy patient is to be fully vaccinated if possible.
- A review of evidence surrounding HCW transmission revealed a relatively low-rate risk of viral transmission overall

### Surgical technique

- Regarding type of tracheotomy, either a percutaneous or open tracheotomy may be performed if clinically indicated. Based on recent evidence [2], outcomes and risk to the patient and healthcare workers appear to be similar for performance of both percutaneous tracheotomy and open tracheotomy [20, 21].

## Caveats

As previously noted, the COVID-19 pandemic has been fraught with a continuously changing situation. While the improved availability of PPE, treatments and both patient and HCW vaccine status may herald a possible light at the end of the tunnel, opposing factors such as more contagious/virulent viral variants and vaccine fears threaten this progress. Such aspects as well as further evidence may lead to further evolution of these and other similar recommendations. Although patient vaccination status and similar aforementioned factors likely affect disease course and health care worker transmission risk in these cases, data is still too limited at this time and further analysis is warranted.

## Summary of changes

The taskforce recommends that it is reasonable to consider tracheotomy after (the greater of) 2 weeks duration of mechanical ventilation or greater than 20 days after symptoms emerge/first positive RT-PCR test. However, we recommend this decision is made on a case-by-case basis based on the overall trajectory of the patient’s outcome and comorbidities whereby performance of a tracheotomy will be more likely to provide a benefit to the patient’s outcome. This should be weighed alongside ICU capacity, resources and surgical considerations. Open surgical and percutaneous techniques appear to have similar safety and outcome profiles. These recommendations are not meant to supersede local governmental or institutional guidelines. See Table 1 for a summary of the main changes comparing the previous and current version. See Table 2 for a summary of the current recommendations based on COVID RT-PCR positivity.

**Table 1:** Summary of changes comparing previous and current taskforce recommendations

	Previous taskforce recommendation	Current (revised) taskforce recommendation
**Covid transmission risk**	Suspected to be low only after the patient tested negative with RT-PCR (regardless of time/duration of symptoms)	Evidence suggests being low if > 20 days from initial symptom onset/initial positive COVID RT-PCR test and > 2 weeks of mechanical ventilation.
**Vaccination status**	N/A	If available, vaccination of surgical/anesthetic/HCW team involved.
**Surgical technique**	Preference for open tracheotomy	Open or percutaneous tracheotomy appear to have similar outcomes and risks to the patient and HCWs involved.

*RT-PCR* reverse transcription polymerase chain reaction, *HCW* healthcare workers

**Table 2:** Summary of recommendations based on RT-PCR COVID-19 positivity

Covid-19 RT-PCR	Current Recommendation
**Positive (< 20 DAYS)**	Recommend against performing a tracheotomy in this group of patients who are potentially still infectious, unless urgent e.g. due to inadequate airway.
**Positive (> 20 DAYS)**	Tracheotomy can be performed if clinically indicated with full aerosol PPE (at least N95 and full face/eye protection for the surgical team).
**Positive (> 90 DAYS)**	Patients should be retested for COVID-19 and if positive, the patient should be considered potentially infectious (for 20 days). If the patient is retested for COVID-19 and if negative, should be treated as COVID-19 negative.
**Negative**	Tracheotomy can be performed if clinically indicated with full PPE (N95 and full face/eye protection for the surgical team).
**Unknown status emergent**	Recommend use of full aerosol PPE including (at least N95 masks with full face/eye protection). Option to use PAPR/N99 equipment or equivalent if available.

*PPE* personal protective equipment, *PAPR* powered air purifying respirator

## Abbreviations

AGMP: Aerosol generating medical procedure
ARDS: Acute respiratory distress syndrome
CDC: Centers for Disease Control and Prevention
CSO-HNS: Canadian Society of Otolaryngology-Head & Neck Surgery
HCW: Health care worker
ICU: Intensive care unit
PPE: Personal protective equipment
PAPR: Powered air purifying respirator
RT-PCR: Reverse transcription polymerase chain reaction
